# Supplementary figures and images for: Endoplasmic reticulum stress activates human IRE1α through reversible assembly of inactive dimers into small oligomers
Source: eLife. 2022 Jun 22;11:e74342. doi: 10.7554/eLife.74342 (PMC9217129; doi:10.7554/eLife.74342)

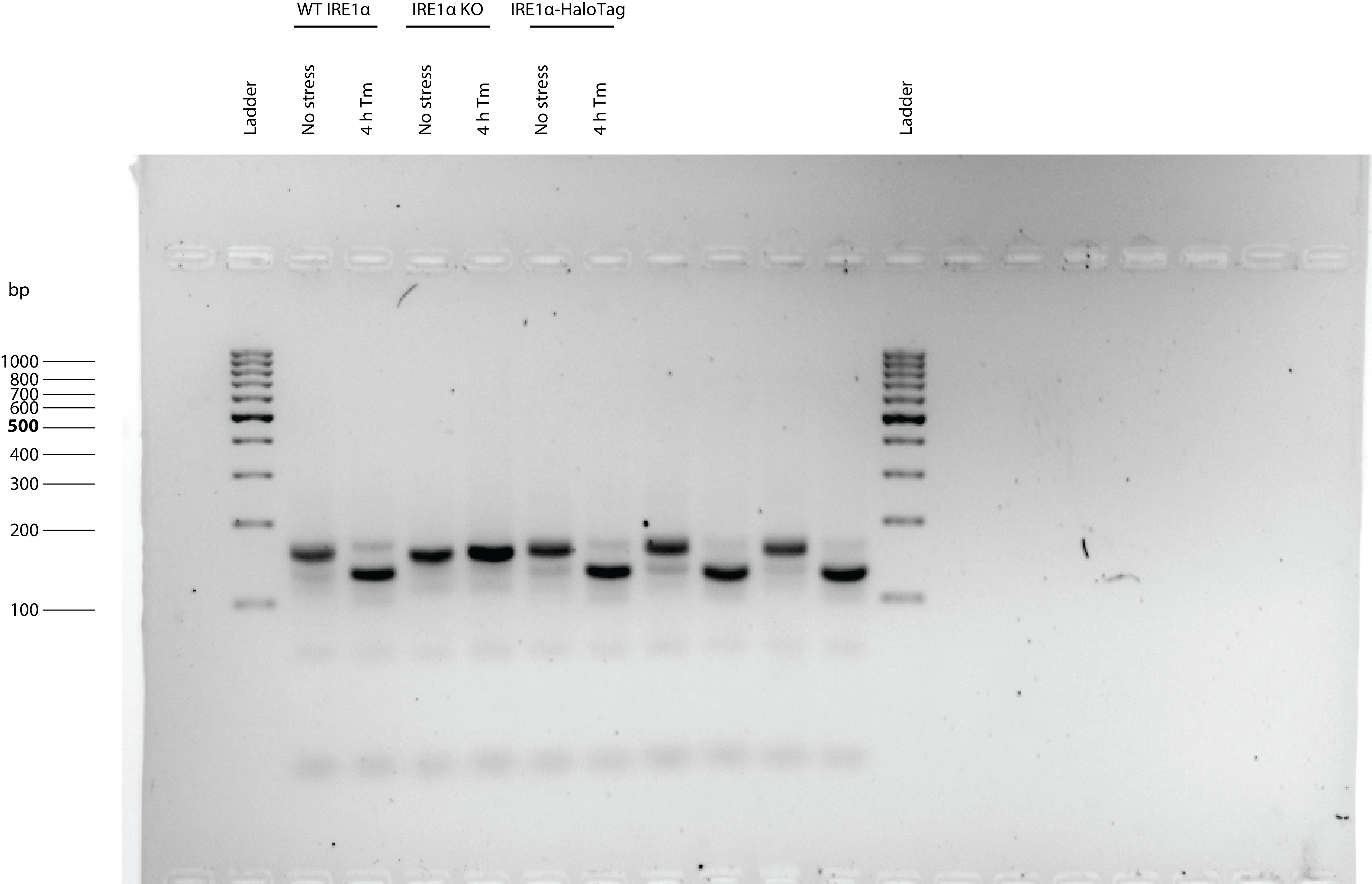

Supplement: Figure 1—source data 1. [file elife-74342-fig1-data1.zip › Figure1-source_data_1.png]

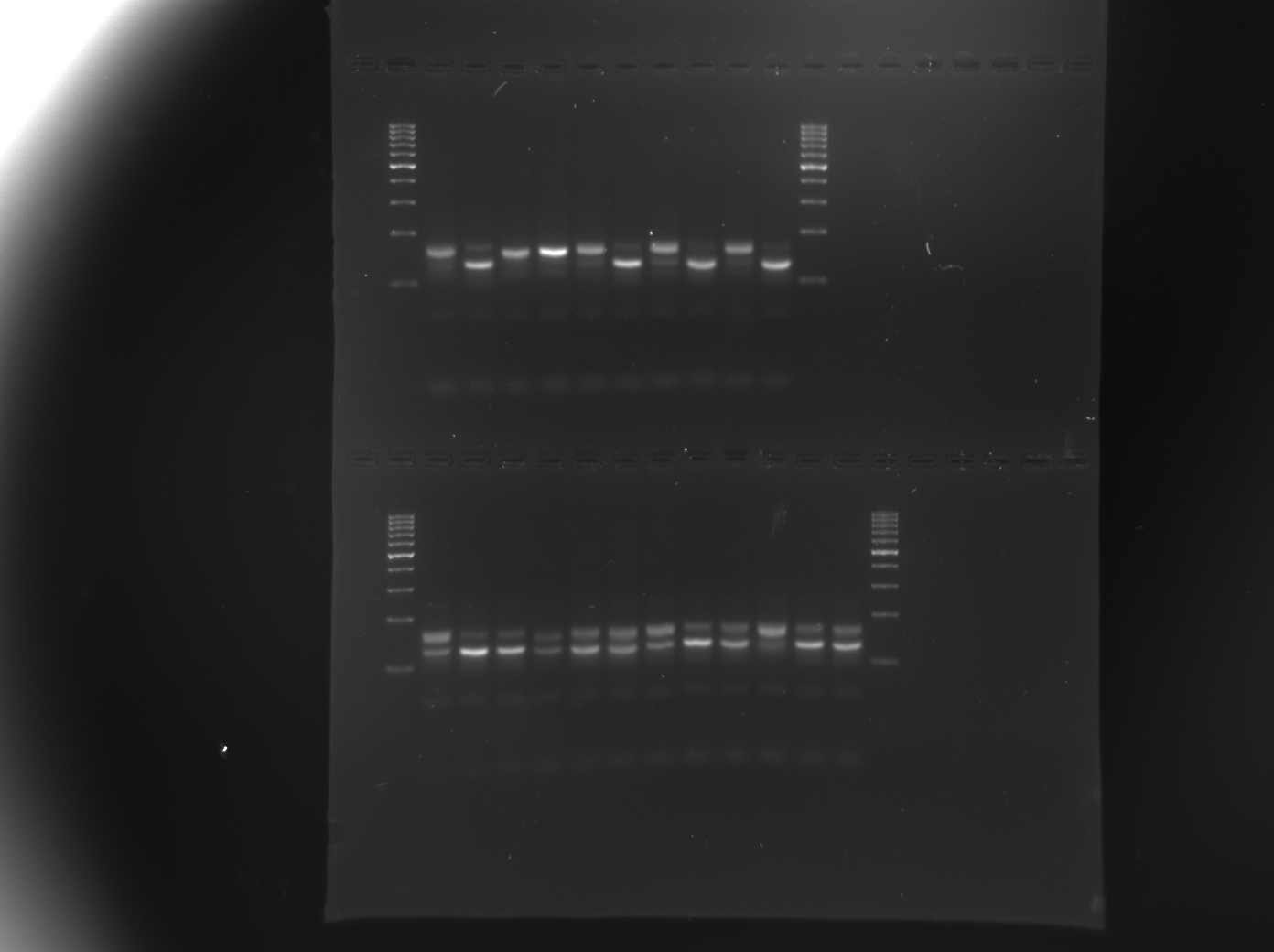

Supplement: Figure 1—source data 2. [file elife-74342-fig1-data2.zip › Figure1-source_data_2.tif]

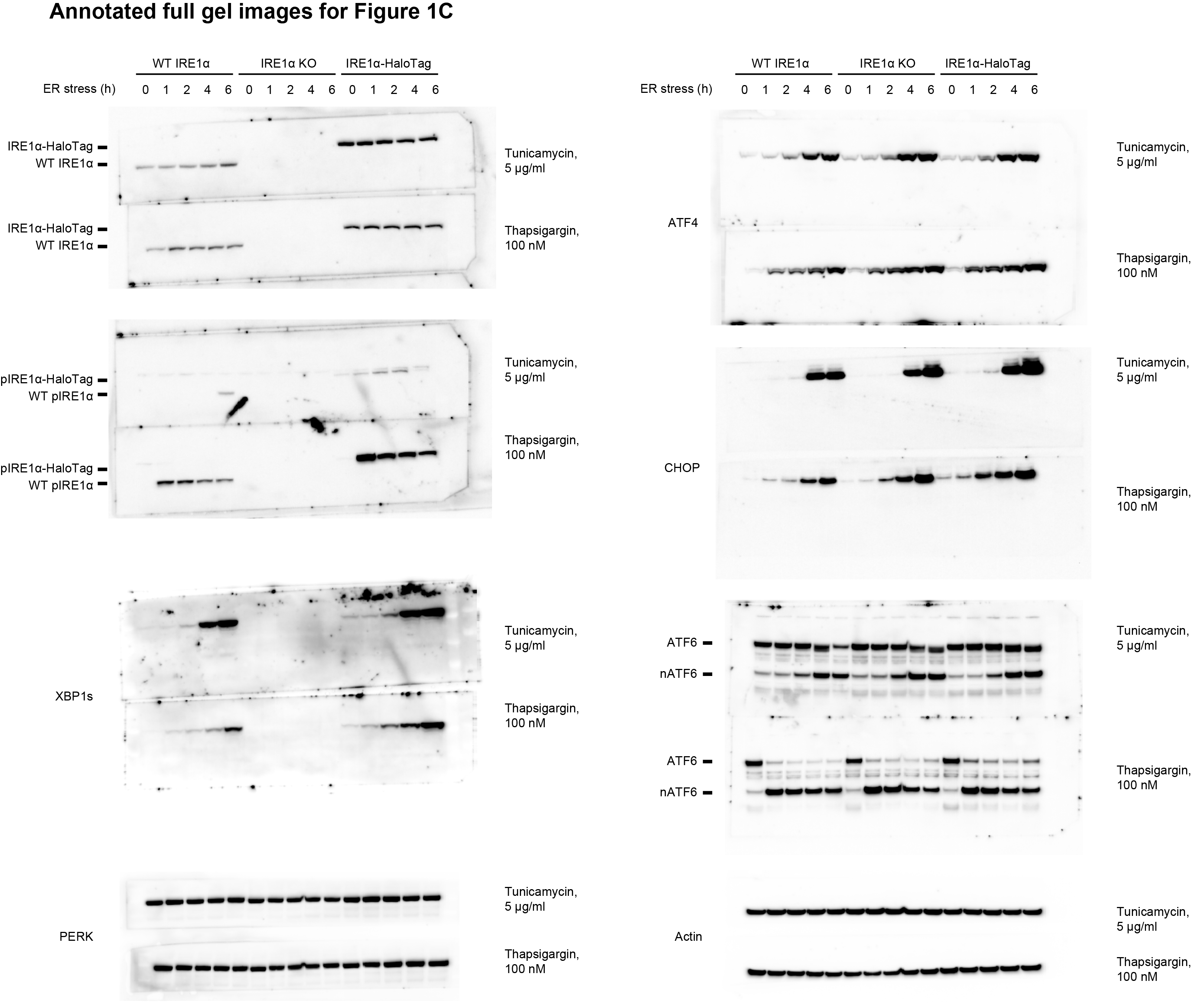

Supplement: Figure 1—source data 3. [file elife-74342-fig1-data3.zip › Figure1-source_data_3-AnnotatedGelsPanelC.tif]

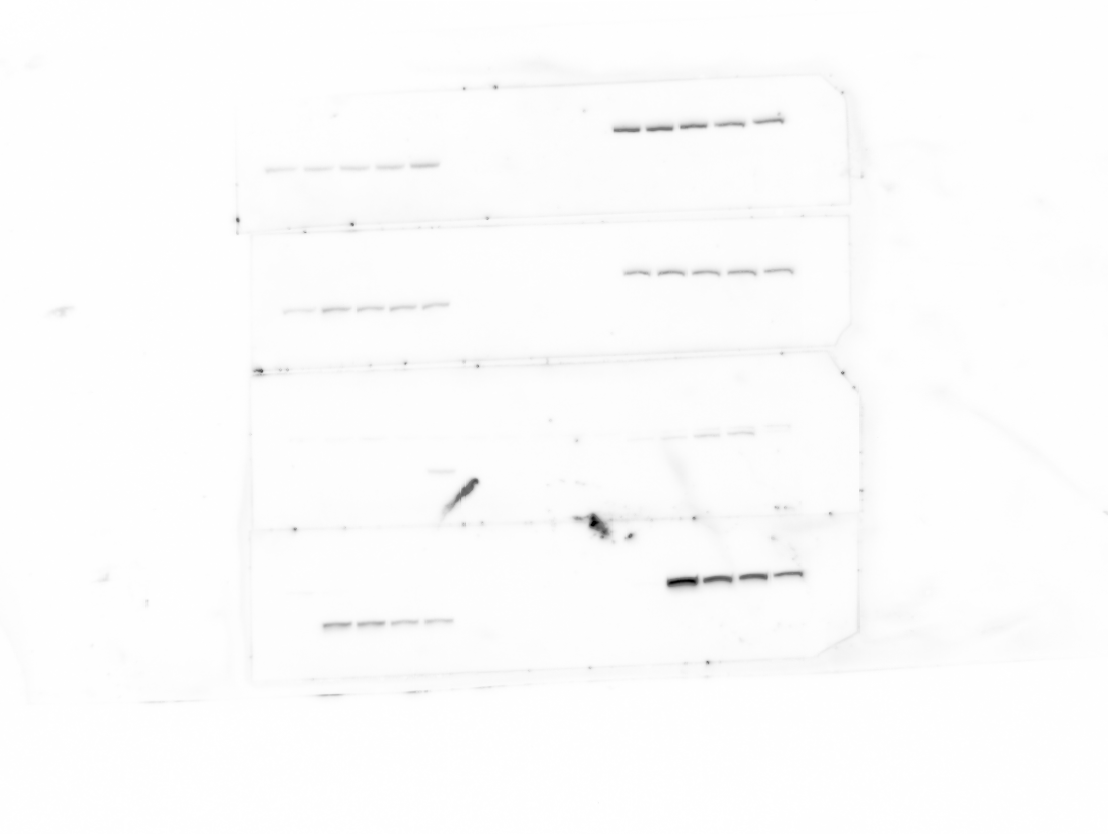

Supplement: Figure 1—source data 4. [file elife-74342-fig1-data4.zip › Figure1-source_data_4-IRE1_pIRE1.tif]

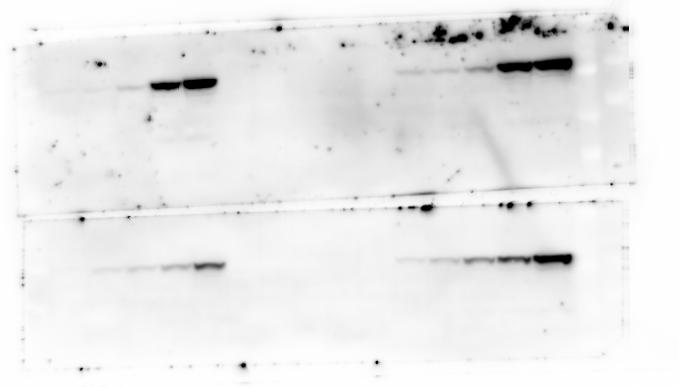

Supplement: Figure 1—source data 5. [file elife-74342-fig1-data5.zip › Figure1-source_data_5-XBP1.tif]

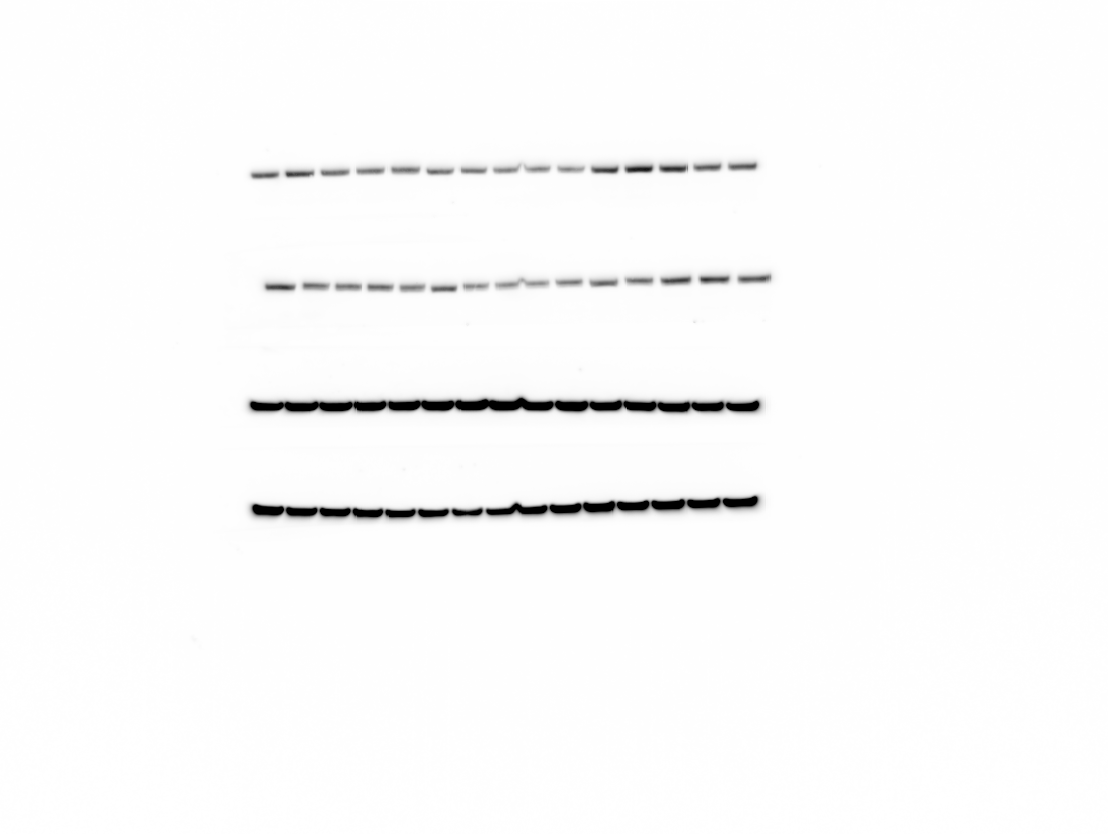

Supplement: Figure 1—source data 6. [file elife-74342-fig1-data6.zip › Figure1-source_data_6-PERK_Actin.tif]

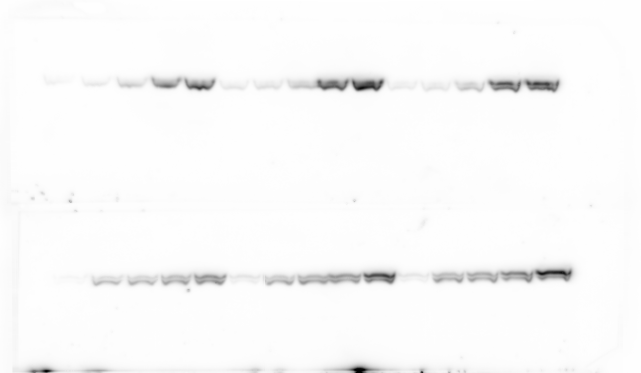

Supplement: Figure 1—source data 7. [file elife-74342-fig1-data7.zip › Figure1-source_data_7-ATF4.tif]

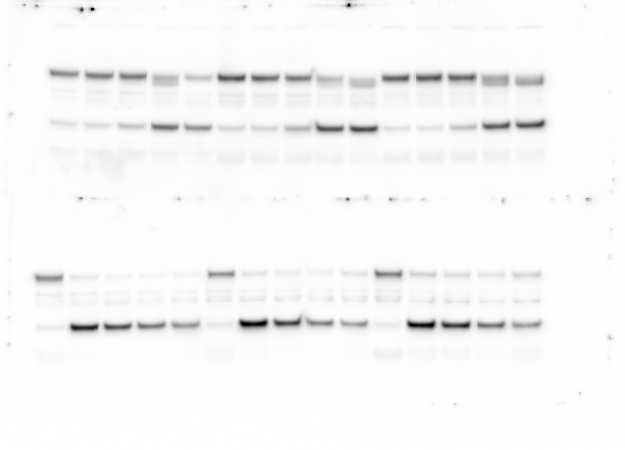

Supplement: Figure 1—source data 8. [file elife-74342-fig1-data8.zip › Figure1-source_data_8-ATF6.tif]

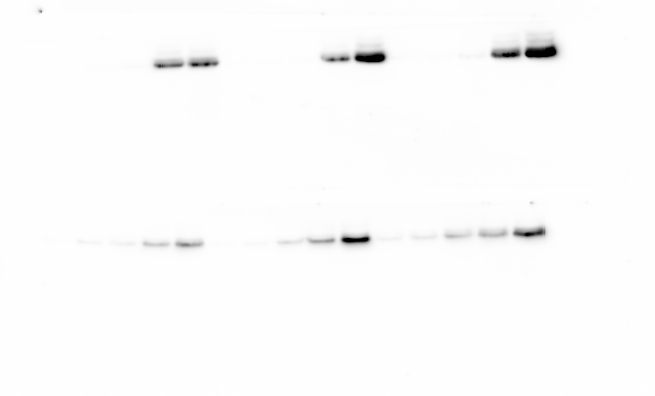

Supplement: Figure 1—source data 9. [file elife-74342-fig1-data9.zip › Figure1-source_data_9-CHOP.tif]

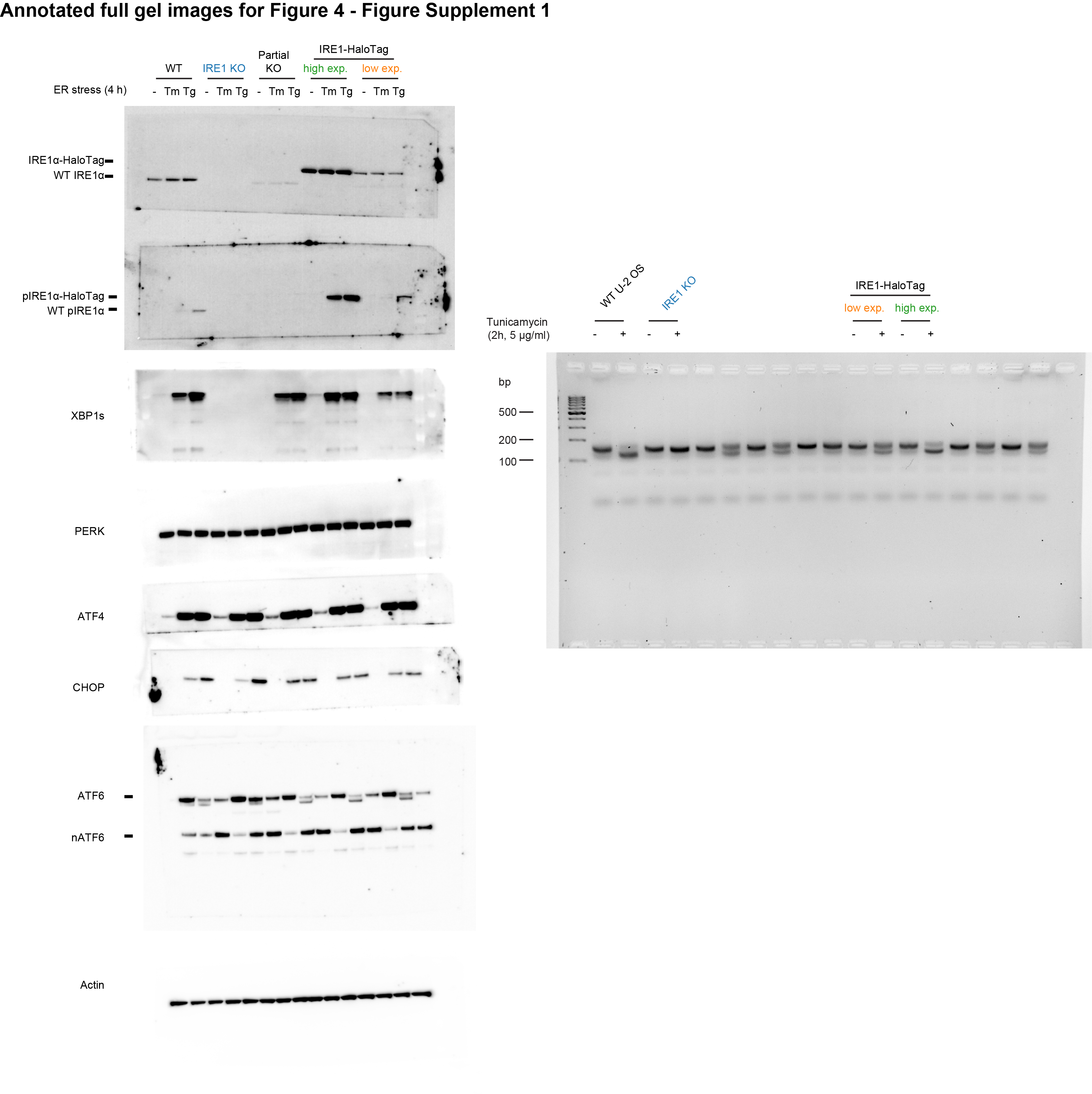

Supplement: Figure 1—figure supplement 1—source data 1. [file elife-74342-fig1-figsupp1-data1.zip › Figure1-Figure_Supplement_1-source_data_1-AnnotatedGels.tif]

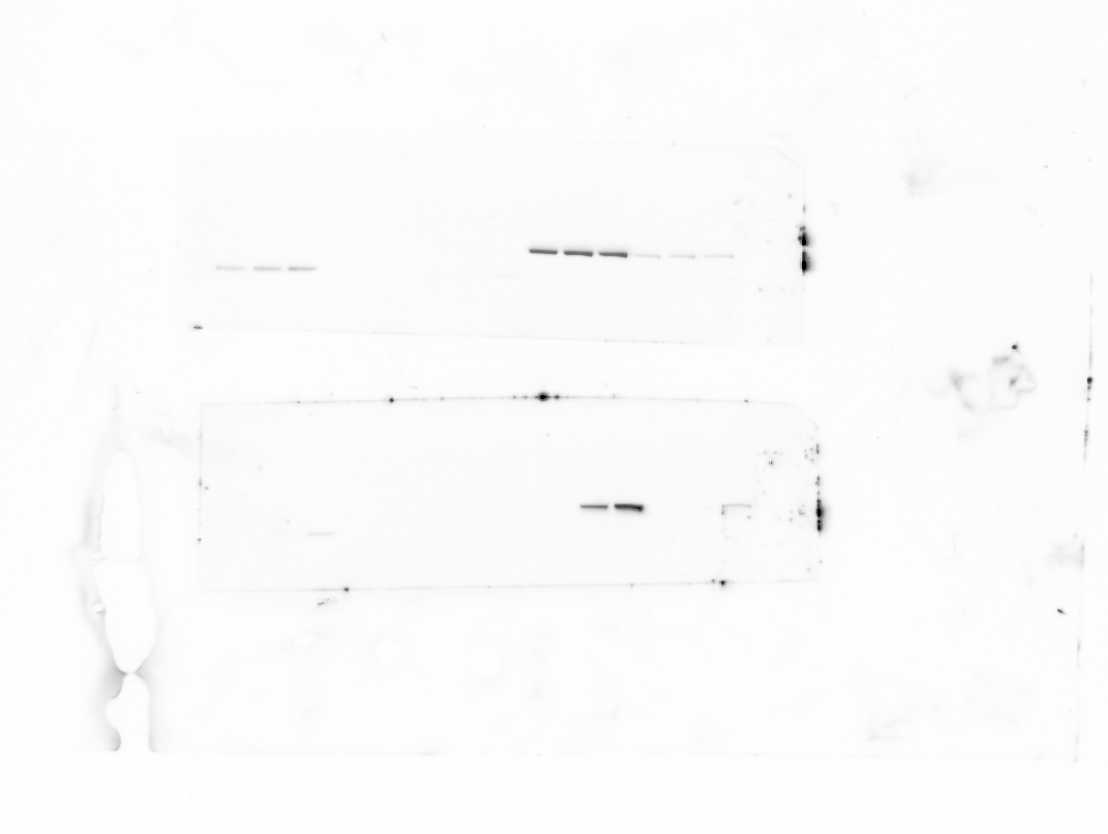

Supplement: Figure 1—figure supplement 1—source data 2. [file elife-74342-fig1-figsupp1-data2.zip › Figure1-Figure_Supplement_1-source_data_2-IRE1-pIRE1.tif]

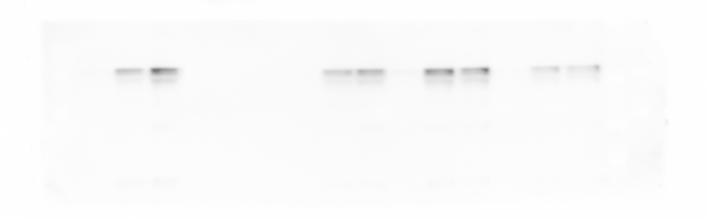

Supplement: Figure 1—figure supplement 1—source data 3. [file elife-74342-fig1-figsupp1-data3.zip › Figure1-Figure_Supplement_1-source_data_3-XBP1s.tif]

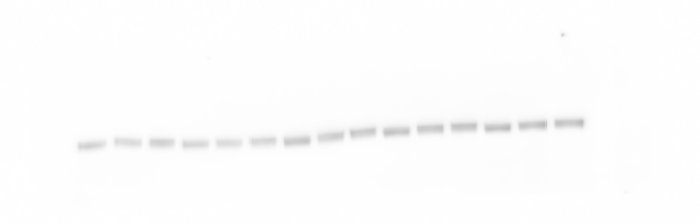

Supplement: Figure 1—figure supplement 1—source data 4. [file elife-74342-fig1-figsupp1-data4.zip › Figure1-Figure_Supplement_1-source_data_4-PERK.tif]

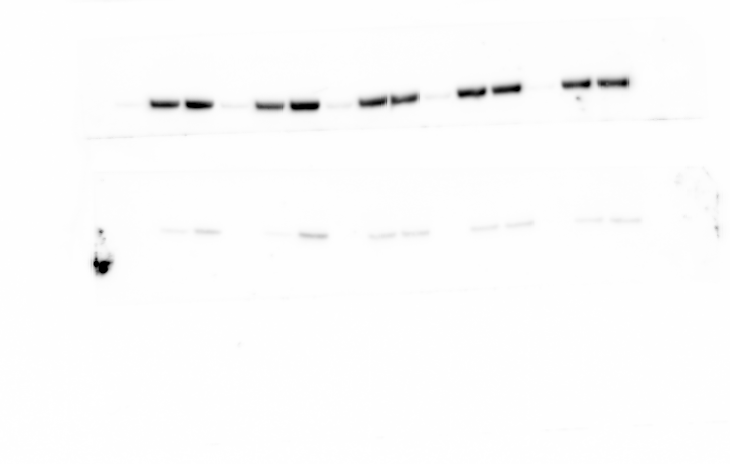

Supplement: Figure 1—figure supplement 1—source data 5. [file elife-74342-fig1-figsupp1-data5.zip › Figure1-Figure_Supplement_1-source_data_5-ATF4-CHOP.tif]

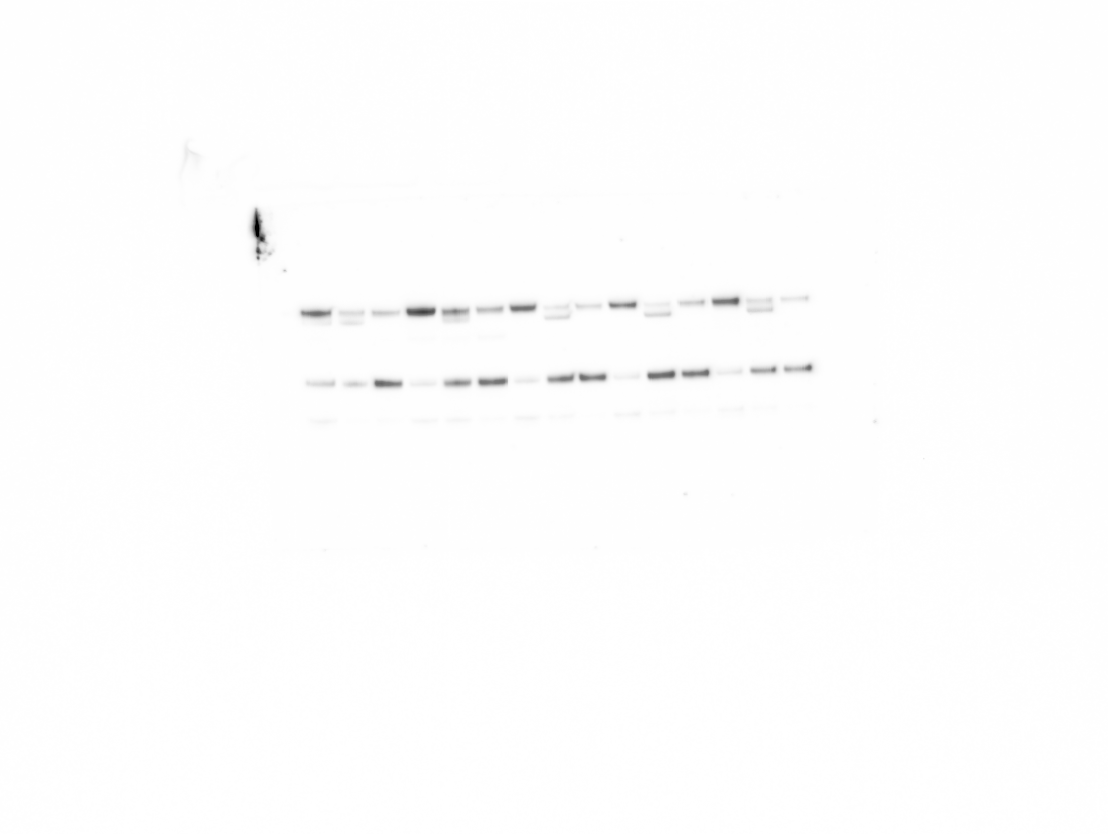

Supplement: Figure 1—figure supplement 1—source data 6. [file elife-74342-fig1-figsupp1-data6.zip › Figure1-Figure_Supplement_1-source_data_6-ATF6.tif]

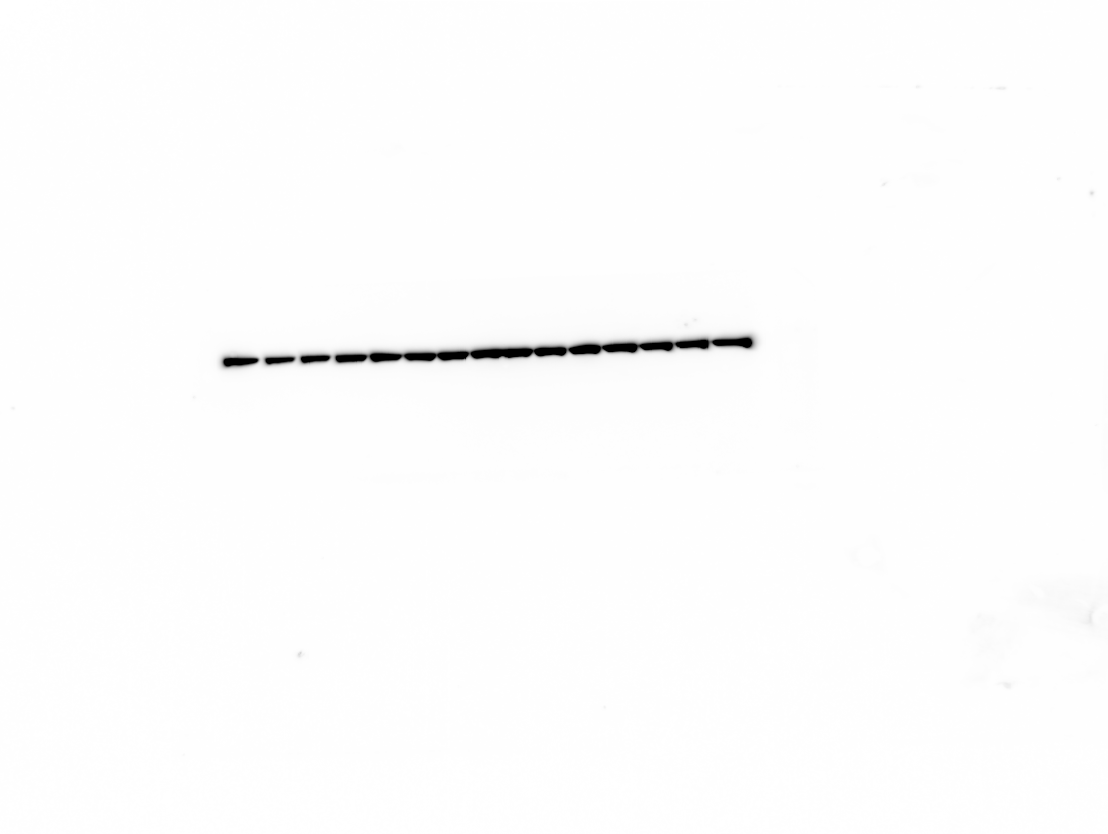

Supplement: Figure 1—figure supplement 1—source data 7. [file elife-74342-fig1-figsupp1-data7.zip › Figure1-Figure_Supplement_1-source_data_7-Actin.tif]

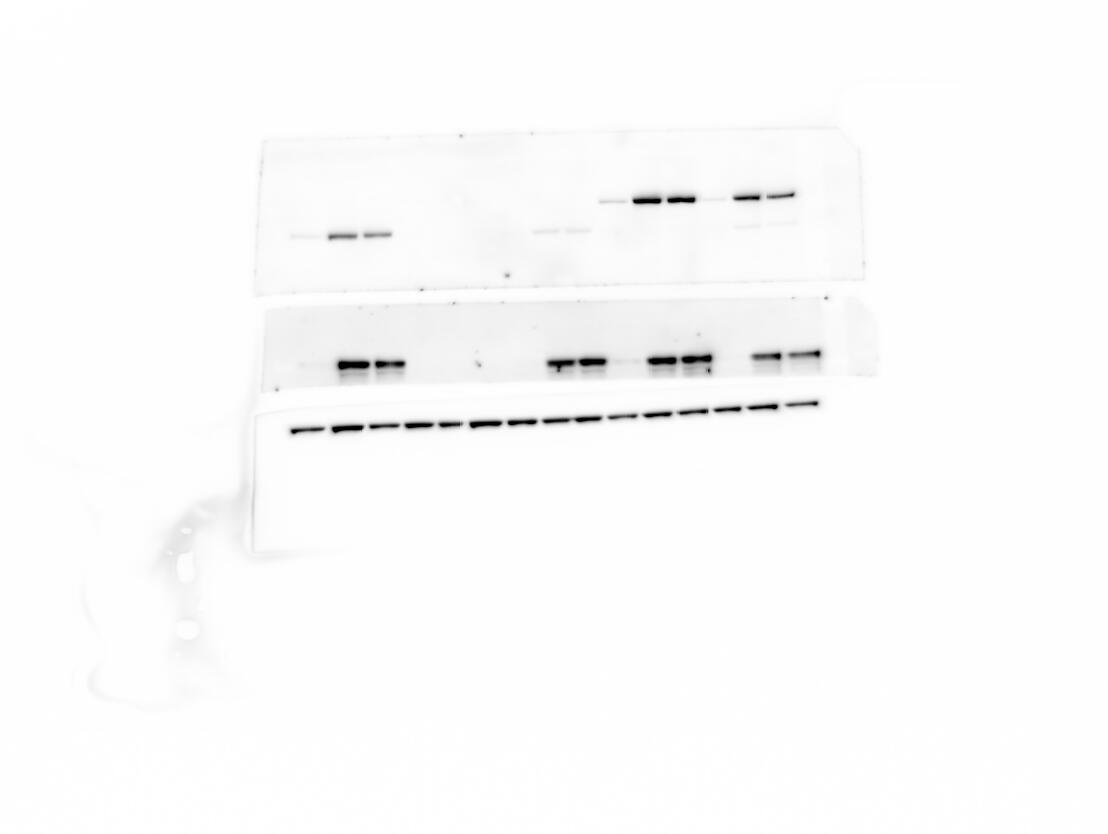

Supplement: Figure 4—figure supplement 1—source data 1. [file elife-74342-fig4-figsupp1-data1.zip › Figure 4—figure supplement 1—source data 1.jpg]

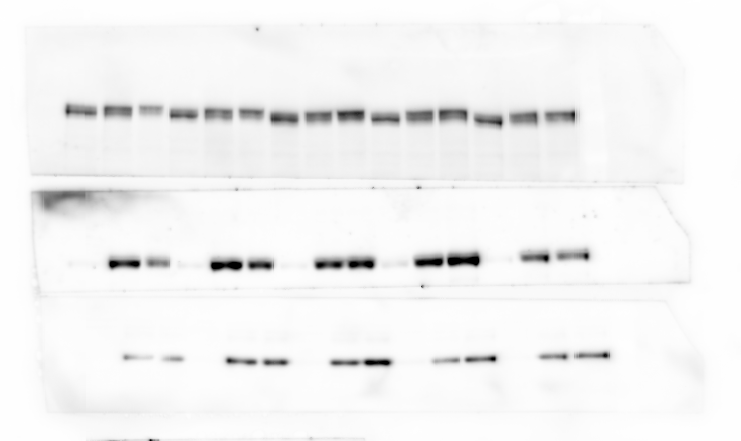

Supplement: Figure 4—figure supplement 1—source data 2. [file elife-74342-fig4-figsupp1-data2.tif]

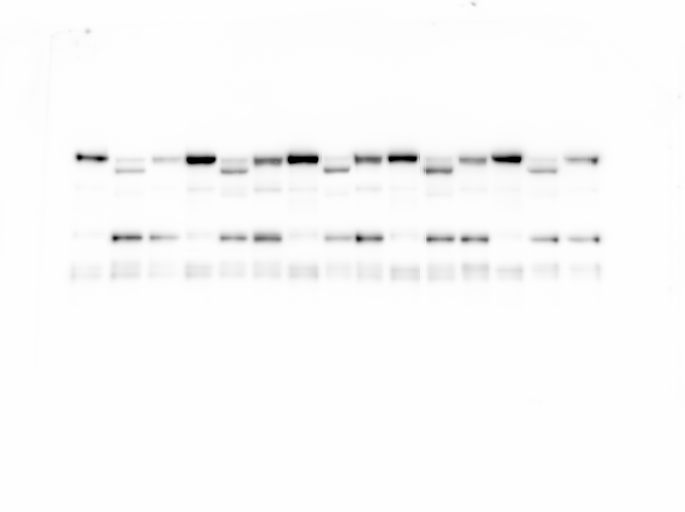

Supplement: Figure 4—figure supplement 1—source data 3. [file elife-74342-fig4-figsupp1-data3.tif]
