# Supplementary material for: Endoplasmic reticulum stress activates human IRE1α through reversible assembly of inactive dimers into small oligomers
Source: eLife. 2022 Jun 22;11:e74342. doi: 10.7554/eLife.74342 (PMC9217129; doi:10.7554/eLife.74342)
Supplement: Supplementary file 1. [file elife-74342-supp1.docx]

# Supplementary File 1

**Detailed information for all plasmids used in this study.**

| Plasmid ID | Plasmid name | Resistance | Description |
| --- | --- | --- | --- |
| pPW3754 | SpCas9 and gRNA targeting the C-terminus of HsIRE11 | Ampicillin | Expression of human codon-optimized SpCas9 and gRNA targeting the C-terminus of human IRE1. |
| pPW3755 | HDR-HsIRE1a-10xGS-HaloTag | Ampicillin + G418 (mammalian) | Complete HDR template for cloning a C-terminal 10xGS-HaloTag into human IRE1alpha. Should be co-transfected with a plasmid encoding the corresponding gRNA and Cas9. Contains a mutated PAM site to ensure that the genome is no longer cut after a successful HDR event. |
| pPW3756 | CMVd3-ERmembrane-HaloTag-KKMP | Kanamycin | Construct for low-level transient expression of a single HaloTag protein targeted to the ER membrane. ER targeting is achieved by an IRE1-derived signal peptide and TM helix, with a C-terminal KKMP ER retention signal. Expression is driven by the heavily truncated CMVd3 promoter. |
| pPW3757 | CMVd3-ERmembrane-2xHaloTag-KKMP | Kanamycin | Construct for low-level transient expression of two tandem HaloTag proteins targeted to the ER membrane. |
| pPW3758 | CMVd3-HsIRE1-HaloTag | Kanamycin | Construct for low-level transient expression of full-length HsIRE1 with a C-terminal HaloTag, with the exact same 10x GS linker sequence as that in pPW3755. Expression is driven by the heavily truncated CMVd3 promoter. |
| pPW3759 | CMVd3-HsIRE1deltaLD-HaloTag | Kanamycin | Construct for low-level transient expression of delta-lumenal domain HsIRE1 with a C-terminal HaloTag. |
| pPW3760 | CMVd3-HsIRE1-K599A_KinaseDead-HaloTag | Kanamycin | Construct for low-level transient expression of kinase-dead (K599A) HsIRE1 with a C-terminal HaloTag. |
| pPW3761 | CMVd3-HsIRE1(WLLI-GSGS)[359-362]-HaloTag | Kanamycin | Construct for low-level transient expression of HsIRE1 with a lumenal domain IF2 mutation (WLLI-GSGS) [359-362] with a C-terminal HaloTag. |
| pPW3762 | CMVd3-HsIRE1(K121Y)-HaloTag | Kanamycin | Construct for low-level transient expression of HsIRE1 with a lumenal domain IF1 mutation (K121Y) with a C-terminal HaloTag. |
| pPW3763 | CMVd3-HsIRE1dLKR-HaloTag | Kanamycin | Construct for low-level transient expression of delta-LKR HsIRE1 with a C-terminal HaloTag. |
| pPW3781 | CMVd3-ERmembrane-GST-HaloTag-KKMP | Kanamycin | Construct for low-level transient expression of a single GST-fused HaloTag protein targeted to the ER membrane. The GST fusion causes this to be a constitutive dimer. |
| pPW3783 | CMVd3-ERmembrane-GST-2xHaloTag-KKMP | Kanamycin | Construct for low-level transient expression of a tandem GST-fused HaloTag protein targeted to the ER membrane. The GST fusion causes this to be a constitutive dimer of dimers, i.e. an effective tetramer of HaloTag. |
